# Peptide and Lipid Modulation of Glutamatergic Afferent Synaptic Transmission in the Solitary Tract Nucleus

**DOI:** 10.3389/fnins.2012.00191

**Published:** 2013-01-10

**Authors:** Michael C. Andresen, Jessica A. Fawley, Mackenzie E. Hofmann

**Affiliations:** ^1^Department of Physiology and Pharmacology, Oregon Health and Science UniversityPortland, OR, USA

**Keywords:** solitary tract nucleus, neuropeptides, capsaicin, TRPV1, vagal afferents

## Abstract

The brainstem nucleus of the solitary tract (NTS) holds the first central neurons in major homeostatic reflex pathways. These homeostatic reflexes regulate and coordinate multiple organ systems from gastrointestinal to cardiopulmonary functions. The core of many of these pathways arise from cranial visceral afferent neurons that enter the brain as the solitary tract (ST) with more than two-thirds arising from the gastrointestinal system. About one quarter of ST afferents have myelinated axons but the majority are classed as unmyelinated C-fibers. All ST afferents release the fast neurotransmitter glutamate with remarkably similar, high-probability release characteristics. Second order NTS neurons receive surprisingly limited primary afferent information with one or two individual inputs converging on single second order NTS neurons. A- and C-fiber afferents never mix at NTS second order neurons. Many transmitters modify the basic glutamatergic excitatory postsynaptic current often by reducing glutamate release or interrupting terminal depolarization. Thus, a distinguishing feature of ST transmission is presynaptic expression of G-protein coupled receptors for peptides common to peripheral or forebrain (e.g., hypothalamus) neuron sources. Presynaptic receptors for angiotensin (AT1), vasopressin (V1a), oxytocin, opioid (MOR), ghrelin (GHSR1), and cholecystokinin differentially control glutamate release on particular subsets of neurons with most other ST afferents unaffected. Lastly, lipid-like signals are transduced by two key ST presynaptic receptors, the transient receptor potential vanilloid type 1 and the cannabinoid receptor that oppositely control glutamate release. Increasing evidence suggests that peripheral nervous signaling mechanisms are repurposed at central terminals to control excitation and are major sites of signal integration of peripheral and central inputs particularly from the hypothalamus.

The nervous system makes a unique contribution to the regulatory mechanisms that help to preserve homeostatic integrity in the face of constantly changing demands. The central nervous system (CNS) serves as a key integrator and coordinator across multiple organ systems and tissues. The CNS and its peripheral neural partners are not only capable of rapidly responding over great distances to coordinate organ function but they also have the capacity to remodel chronically. By orchestrating visceral organ functions, the CNS helps to foster effective system-wide control over the conditions maintaining cellular homeostasis. The homeostatic palate of regulated “states” spans vital energy, temperature, ionic, and metabolic status (Schwartz et al., 2000; Nakamura and Morrison, 2008; Dallman, 2010; Grill and Hayes, 2012). Organism-wide balance matches critical processes mediating environmental exchange (at the skin, lung, and gastrointestinal tract) with the appropriate transportation (cardiorespiratory and gastrointestinal). The regulatory challenge, of course, is dynamic in that acute changes in organ conditions require speedy adjustments but long term, chronic changes require the capacity to adapt to sustained changes. Even in normal physiological circumstances, a new state can require adjustments in the performance characteristics of neural control pathways to accommodate these normal changes. For example, chronic aerobic fitness training often restructures the heart so that the resting stroke volume is increased but this new state requires complementary changes in neural regulation around a new, lower resting heart rate (Jackson et al., 2005; Higa-Taniguchi et al., 2009; Cavalleri et al., 2011). All circumstances require homeostatically similar outcomes that provide a consistent cellular milieu of blood gases, nutrients, and waste removal. However, frank dysregulation increasingly is recognized as an integral part of pathological states such as metabolic syndrome in which multiple systems become compromised or even fail (Alberti et al., 2005, 2006; Perez-Tilve et al., 2006). The aim of the review is to focus on the building blocks of neural synaptic transmission that determine and modify the performance of these neural pathways.

One fundamental aspect essential to understanding homeostatic and pathologic complexities is an appreciation of normal regulation and the framework and structure of interactions across organ systems. The goal of this chapter is to focus on the neural signaling interface between incoming information about visceral status and the initiation of CNS regulatory responses. For organ regulation, this afferent-CNS interconnection occurs largely at the caudal third of the nucleus of the solitary tract (NTS). The NTS is a major gateway for cranial visceral afferent information (Andresen and Kunze, 1994; Andresen and Paton, 2011) and, as such, is a unique focus of visceral organ information in the CNS. This review summarizes the fundamental building blocks for the processing of afferent signals at the NTS as part of overall integration and illustrates that information processing at this site goes well beyond a simple relay and broadcast of visceral signals. Recent work suggests that, across multiple organ systems, all circuits involving the NTS appear to utilize similar basic communication mechanisms – *mechanisms in common*. In contrast, however, other aspects offer *mechanisms of distinction* such as ligand-gated ion channels and G-protein coupled receptor (GPCR)-based systems that may distinguish particular pathways.

## Indistinct Neighborhoods

Overall, the NTS is quite diverse. Structurally, the NTS has regions distinguished cytoarchitecturally by morphological features (Loewy and Burton, 1978; Kalia and Sullivan, 1982). However, the distribution of cranial visceral afferent axons and terminations poorly conform to this cytoarchitectural topography, particularly rostro-caudally. Thus, despite input fibers that arise from distinctly different visceral organs (e.g., gastrointestinal system, heart, blood vessels, lung, or airways), these various afferents distribute broadly within NTS and are indistinct in location and morphology. This diversity and ambiguity of distribution extends to neurochemical phenotypes of NTS neurons – glutamatergic, γ amino-butyric acid (GABA) synthesizing, catecholamine (CA) synthesizing, pro-opiomelanocortin (POMC), and other unique neurons (Andresen and Kunze, 1994; Andresen et al., 2007a; Wan et al., 2008). One early idea held that particular afferent inputs might specifically contact histologically defined sub nuclei within the caudal NTS (Kalia and Mesulam, 1980). Across studies however, multiple methods of tracing markers of cranial afferents from visceral organs to their destinations in the NTS produced “cloud”-like maps that crossed morphological sub regions and, in addition, functional approaches using physiological activation generated regional overlays that were similarly indistinct (Andresen and Paton, 2011). Thus, cardiovascular investigators produced diagrams representing locations of NTS neurons involved in cardiovascular regulation (Ciriello and Calaresu, 1981; Donoghue et al., 1981b; Chan and Sawchenko, 1998; Corbett et al., 2005). Respiratory (Kubin et al., 1991; Haxhiu and Loewy, 1996) or gastrointestinal (Altschuler et al., 1991; Babic et al., 2009) investigators also diagrammed associated functional zones. The results of these targeted distributions, however, are remarkably similar to each other, and the resemblance suggests that these afferent-linked functional regions largely overlap and correspond to anatomic sub regions with fuzzy boundaries and co-mingled neurons of different functions. Such global maps are further confounded by a common cellular feature of NTS neurons – the dendrites of individual NTS neurons extend to reach across considerable distances (Deuchars et al., 2000; Paton et al., 2000; Kubin et al., 2006). Thus, the synaptic locale of single neurons is ambiguous and often best attributed to multiple anatomical sub nuclei. This aspect of cellular anatomy further blurs the spatial boundaries for functional groups (e.g., baroreceptive vs. gastrointestinal regions) as pharmacological targets. Lastly, important motor neurons within the dorsal motor nucleus (DMN) spread dendrites into anatomically defined NTS sub regions and some synaptic terminals from vagal afferents directly contact DMN cell processes (Rinaman et al., 1989).

## Afferent Connections at NTS

The physical wiring pathways for neural regulation begin with the visceral primary afferents of cranial nerves and their physical contacts on NTS second order sensory neurons (Andresen and Kunze, 1994; Craig, 2002; Andresen and Paton, 2011). However, in most vital organs, visceral sensory primary afferents arise from *dual* sources. Cranial visceral afferents have cell bodies chiefly in the nodose and petrosal ganglia and send their afferent axons into the NTS. A second afferent source arises from spinal visceral afferents which have cell bodies located in the dorsal root ganglia but their axons enter the CNS at the spinal cord dorsal horn. Both cranial and spinal visceral sensory neurons include myelinated (mostly Aδ) and unmyelinated subtypes and, for most organs, the slowly conducting C-type afferents greatly outnumber the myelinated class of afferents by 8–10-fold regardless of their cranial or spinal destination (Agostoni et al., 1957; Andresen et al., 1978; Sant’Ambrogio, 1987; Kubin and Davies, 1995). This cranial information flows mostly through the IXth and Xth cranial nerves to form the first step of pathways which are largely concentrated in the brainstem. These brainstem pathways have a structure that feeds back to regulate the organs from which those afferents originated. Spinal visceral primary afferents often follow sympathetic nerve trunks to reach their cell bodies within the dorsal root ganglia. This spinal information may contribute to nociceptive signaling within the spinal cord and less directly influences visceral organ regulation (Foreman, 1999, 2000; Gamboa-Esteves et al., 2001). The synapses from cranial visceral primary afferents define second order afferent neurons within the NTS, but the final projections of the axons of most second order NTS neurons are less certain. In fact, it is quite likely that many second order NTS neurons have multi-functional axon architectures in which branches from single neurons serve local interneuron functions and other branches project to targets outside of the NTS. Many of these pathways may be relatively inactive under normal circumstances since C-fiber afferent have high thresholds for sensory activation and do not contribute to resting control in autonomic regulation and satiety (Aars et al., 1978; Ritter, 2004).

## Limited Afferent Convergence

Early work established that cranial visceral afferent responsive neurons were located in common sub regions within the NTS. Given the close proximity of different afferent terminals, a key question was whether afferent information from multiple organs impinged on single NTS neurons. The cervical vagus nerve contains mostly afferent axons. Despite its >10,000 cranial visceral afferents axons, maximal electrical activation of the vagal trunk generally triggered responses consistent with a single input activating NTS neurons (Agostoni et al., 1957). Multiple assessments of the potential for convergence of afferents from separate nerves onto single neurons suggested that <15% of individual NTS neurons have multiple afferent inputs (Donoghue et al., 1981b, 1985; Paton, 1998). Even under intense stimulation, arterial baroreceptor activation did not trigger responses by converging onto NTS neurons activated by cardiac afferents (Silva-Carvalho et al., 1998; Seagard et al., 1999). Tests in unit recordings with multiple converging inputs showed that the additional inputs arrived via polysynaptic paths – that is, indirect relays rather than direct terminations of multiple different afferents on single second order NTS neurons (Zhang and Mifflin, 1995; Mifflin, 1996). Another remarkable feature of convergence is that afferent inputs are segregated by their axon fiber type. NTS neurons receiving C-fiber inputs do not also directly receive myelinated afferent inputs (Donoghue et al., 1981a; Doyle et al., 2002; Jin et al., 2004b; Andresen and Peters, 2008). As outlined below one of the key phenotypic markers for the unmyelinated afferent axons is the Transient receptor potential vanilloid type 1 (TRPV1). TRPV1+ afferents do not mix with TRPV1− afferents at medial NTS neurons so that TRPV1 afferents are – a *mechanism of distinction* (Peters et al., 2010, 2011; Shoudai et al., 2010) and we thus will refer to the receiving NTS neurons as TRPV1+. Overlapping global afferent distribution maps dispel the earliest notions of dedicated physical localities as centers of dedicated function and instead suggest that there is a close physical proximity of very different afferent circuits destined to regulate very different functions (e.g., gastrointestinal adjacent to airway afferent receiving neurons). At the level of individual neurons, the results demonstrate highly focused organization of pathways that travel nearby but are separated by discrete synaptic connections – a *mechanism of distinction*. This separation importantly preserves functionally separate but parallel neural pathways. Recognition of these fundamental structural features may guide our understanding of the basic mechanisms that underlie the neural regulation of visceral organs and may help optimize therapeutic approaches during ongoing dysregulation or pathology.

## NTS Cellular Diversity Across Circuit Roles

If the afferent-NTS interface is relatively simple but isolated from others, what features distinguish different functional pathways? The NTS is well known for its phenotypic diversity. One major difference is reflected in the neurotransmitter synthesis capacity or neurochemical characteristics of these neurons – a form of cellular phenotype. The neurotransmitters themselves of course are one major cellular difference. At the basics of signaling, central reflex circuits constantly balance excitation with inhibition in propagating signals through networks (Sabatini and Regehr, 1999; Abbott and Regehr, 2004; Semyanov et al., 2004). As elsewhere in the CNS, excitation relies most importantly on glutamate as a neurotransmitter while the primary inhibitory neurotransmitter is GABA.

The relay of excitation from cranial visceral afferents requires NTS neurons of an excitatory neurochemical phenotype. The evidence for excitatory glutamate includes the expression of the vesicular glutamate transporter in NTS neurons including vGlut2 as part of their neurochemical profile (Stornetta et al., 2002; Moechars et al., 2006; Affleck et al., 2012). A selective knockout of vGlut2 is perinatal lethal with suggestions of respiratory failure and such early fatality is consistent with an absolute requirement for this key glutamatergic mechanism (Moechars et al., 2006). NTS projection neurons convey afferent information to key targets beyond NTS including the paraventricular nucleus of the hypothalamus (PVN) via glutamate release (Affleck et al., 2012).

The counterbalance to excitation lies largely in the GABAergic neurons that are interspersed throughout the NTS (Izzo et al., 1992; Chan and Sawchenko, 1998; Stornetta and Guyenet, 1999; Fong et al., 2005) that express the key isoforms of synthetic enzymes, glutamate decarboxylases (GAD), GAD65 and GAD67. GABAergic NTS neurons play well-established, specific roles in respiratory and gastrointestinal regulation. In respiration, GABAergic pump cells are second order NTS neurons which project from NTS to the ventrolateral medulla and the pons but these also serve as local interneurons via recurrent collaterals within the commissural nucleus to inhibit other second order NTS neurons that receive rapidly adapting stretch receptor inputs (Davies et al., 1987; Ezure and Tanaka, 1996; Kubin et al., 2006). For gastrointestinal pathways, GABAergic inhibitory NTS neurons tonically inhibit the DMN of the vagus (Browning and Travagli, 2010). Regardless of the functional pathway, GABAergic NTS neurons are nearly exclusively second order neurons (Glatzer et al., 2007; Bailey et al., 2008) and 70% receive a single cranial visceral afferent (Bailey et al., 2008). However, the density of GABA releasing contacts from single axons is quite limited at NTS neurons (McDougall and Andresen, 2012). Sustained visceral afferent stimulation activated Fos expression in substantial numbers of GABAergic neurons *in vivo* (Chan and Sawchenko, 1998; Weston et al., 2003). Not surprisingly, blockade of GABAergic signaling in the NTS broadly impacted cardiovascular, respiratory, and gastrointestinal regulation (Andresen and Mendelowitz, 1996; Kubin et al., 2006; Travagli et al., 2006; Kenny, 2011).

Although GABA is an important neurochemical phenotype, substantial numbers of NTS neurons feature unique neurochemical phenotypes that represent smaller, diverse sub populations. For example, 20–35% of the NTS neurons in the caudal-postremal region express the key CA synthetic enzyme tyrosine hydroxylase (Austgen et al., 2009). A subset of CA-NTS neurons (10–20%) may be devoted to cardiovascular regulation (Korner, 2007). CA neurons were the focus of considerable work in the early 1960s (Dahlstrom and Fuxe, 1964) as members of “slow neurotransmitter” neurons hypothesized to be key to long term regulatory processes (Korner, 2007). CA-NTS neurons consist of A_2_ noradrenergic neurons and C_2_ adrenergic neurons and CA-NTS neurons were among the first central CA neurons recognized (Dahlstrom and Fuxe, 1964). The A_2_/C_2_ groups of NTS neurons broadly impact homeostatic regulation (Hollis et al., 2004) including cardiovascular, respiratory, and gastrointestinal systems (Andresen and Kunze, 1994; Saper, 2002). Various afferent stimuli activate CA-NTS neurons which then project extensively to areas including the hypothalamus, amygdala, nucleus accumbens, and DMN of the vagus (Riche et al., 1990; Travagli et al., 2006). C_2_ neurons have markedly heterogeneous responses (Chan and Sawchenko, 1994) and some require rather intense afferent challenges to produce activation of FOS expression (Chan and Sawchenko, 1998), circumstances reminiscent of C-fiber recruitment. Unlike in many other areas of the CNS, CA-NTS neurons do not synthesize GABA (Stornetta and Guyenet, 1999).

## Peptidergic Signaling in NTS

Although much of the signaling within the NTS utilizes release of amino acid or biogenic amines as transmitters (Andresen and Kunze, 1994; Jordan, 2005; Kubin et al., 2006), the broad impact of peptide neurotransmitters is unmistakable with more than two dozen peptides and/or their receptors long implicated (Lawrence and Jarrott, 1996; Zhuo et al., 1997; Chaudhri et al., 2008; Michelini and Stern, 2009). Peptides offer another layer of signal integration since their receptors often target ion channels, frequently are presynaptic on afferent terminals and affect the release of other neurotransmitters. The μ-opiate receptor is particularly widespread in the NTS (Dashwood et al., 1988). Far more restricted examples are vasopressin (AVP; Bailey et al., 2006b) and oxytocin (OT; Peters et al., 2008) receptors which are strongly linked to the hypothalamus. These two peptide receptors oppositely regulate the release of glutamate from solitary tract (ST) afferent terminals (OT facilitates release and AVP inhibits release) on limited numbers of neurons within the NTS. These receptors to hypothalamic neuropeptides represent one of the most common schemes for differential pathway control of NTS neurons. In the hypothalamic pattern, AVP or OT receptors reside presynaptically on different sets of cranial afferent terminals and have been linked to plastic changes brought on by exercise conditioning that changes the reflex performance in specific pathways through NTS (Michelini and Morris, 1999; Michelini, 2007; Michelini and Stern, 2009; Stern et al., 2012). Another peptide, angiotensin, facilitates glutamate release from ST afferent terminals acting through AT1 receptors (Barnes et al., 2003) but is much more widespread including postsynaptic NTS neuron membranes but also presynaptically at symmetric synapses that are presumably GABAergic (Huang et al., 2003).

CA-NTS neurons contain subsets of peptide receptors for cholecystokinin (CCK), ghrelin, and opioids, a pattern found in other NTS neuron subsets, and these peptides modify the behavior of specific pathways. Thus, peptide neurotransmission appears to be key mechanism of differential control. Glutamate release onto CA-NTS neurons within gastrointestinal pathways is modified by peptides acting presynaptically on ST afferents to modify transmission and manage pathway outcomes (Appleyard et al., 2007; Cui et al., 2011, 2012a). Ghrelin and opioids strongly inhibited the presynaptic process of release of afferent glutamate onto many CA-NTS neurons (Cui et al., 2011, 2012a). Although CA-NTS neurons often received multiple cranial afferent inputs, it is not clear whether these represent different, converging sensory modalities, i.e., mixed modality inputs or simply multiple similar ST afferents; Appleyard et al., 2007). The ST afferent inputs to CA-NTS neurons were often CCK sensitive and these second order neurons had prominent expression of A-type potassium currents that suppressed action potential generation in a time dependent fashion and thus provided postsynaptic attenuation of afferent transmission (Appleyard et al., 2007). The multi-system aspect of CA-NTS neurons may offer targets in common that reach across otherwise functionally discrete pathways to alter chronic homeostatic, behavioral, or metabolic states during obesity, hypertension, or stress (Kenny, 2011). Co-localization of more than one neuropeptide receptor as well as localization at pre- and/or postsynaptic elements may differentiate control of information to particular targets and increases the combinations for signal crosstalk, e.g., between opioids and CAs (Sumal et al., 1983; Pickel et al., 1989; Velley et al., 1991; Nirenberg et al., 1995). Thus despite relatively early attention to the presence of CA neurons within the NTS, the mechanisms of peptide action, and interaction remain imprecisely known.

Glucagon-like peptide-1 (GLP-1) is increasingly recognized as an important signaling partner molecule that is present peripherally as well as in the NTS. GLP-1 is released from both the gastrointestinal tract and from a limited population of preproglucagon (PPG) NTS neurons (Larsen et al., 1997). GLP-1 affects energy balance (lowers blood glucose and stimulates insulin secretion) and influences thermogenesis, hunger, and satiety (Kenny, 2011). PPG neuron cell bodies are located in the caudal NTS adjacent to area postrema (Llewellyn-Smith et al., 2011). GLP-1 receptor blockade increases food intake (Alhadeff et al., 2012). Despite this strong connection to feeding behaviors and the mesolimbic reward system, injections of the GLP-1 analog exendin-4 also increases heart rate and blood pressure when delivered peripherally or centrally with substantial evidence for activation of TH neurons within the NTS and elsewhere (Yamamoto et al., 2002). Activation of GLP-1 in many pancreas-projecting vagal motoneurons evokes action potentials and is implicated as targeting potassium and GABAergic currents (Wan et al., 2007). One relatively rarer but important neurochemical phenotypes of NTS neurons is the POMC neuron group linked to feeding behavior (Appleyard et al., 2005; Kenny, 2011). Mutated forms of the POMC and melanocortin-4 receptor genes are associated with severe, early onset obesity (Clement et al., 2002). The POMC NTS neurons together with POMC neurons within the arcuate nucleus of the hypothalamus help to regulate energy homeostasis through widespread limbic, motor, and autonomic projections and are modulated by appetite-regulating hormones such as leptin and glucose (Spiegelman and Flier, 2001; Cone, 2005; Rother et al., 2008; Grill, 2010; Mountjoy, 2010; Kenny, 2011). POMC NTS neurons were widely dispersed but CCK activated FOS expression in a dose graded fashion (Appleyard et al., 2005). POMC NTS neurons received direct ST excitatory inputs which were often facilitated by CCK or inhibited by met-enkephalin, both presynaptic actions. Given the gastrointestinal focus of much of this work, it is less clear whether these peptide signals also may be represented in the signaling of other neighboring pathways such as cardiorespiratory control.

A recurring theme in peptide signaling is the discovery of a peptide through its actions on a peripheral end organ followed by evidence for the same peptide within the CNS including the NTS (Chaudhri et al., 2008; Simpson et al., 2012). Such dual representation at disparate sites (e.g., opioids, somatostatin, or CCK) has generated substantial discussion about whether peripherally released peptides influence reflex control solely via peripheral actions on primary afferents or whether those peptides might penetrate the CNS (Herbert and Saper, 1990; Baptista et al., 2005b, 2007). Similar discussions arise concerning peptides with the potential for dual delivery mechanisms – hormonal secretion into the bloodstream or neural signals delivered through specific fiber contacts. With respect to the caudal NTS, the local blood–brain barrier is relatively porous via fenestrated vascular endothelial cells (Gross et al., 1990, 1991; Broadwell and Sofroniew, 1993; Huang et al., 2003). The important implications demand quite challenging, additional comprehensive work to resolve these mechanisms, especially with regard to pathway performance changes during pathophysiological states.

## Destinations

Cellular themes of neurotransmission appear to be commonly repeated across different neurochemical phenotypes of NTS second order neurons. The nature of the functional outcome of actions at NTS neurons, however, will be determined by the destination of its axon. Anterograde and retrograde labeling studies indicate substantial connectivity, often bidirectional, between the caudal NTS and most of the CNS (Herbert et al., 1990; Spyer, 1990; Hermes et al., 2006; Geerling et al., 2010; Rinaman, 2010; Alheid et al., 2011; Alhadeff et al., 2012). Such projections often are part of bidirectional communication with other CNS integrative regions including the paraventricular and lateral nuclei of the hypothalamus, rostral ventrolateral medulla, caudal raphe nuclei, the A5 cell group, and the area postrema (Loewy, 1990). This contrasts with the relatively discrete patterns of axons to and from PVN neurons of the hypothalamus or the caudal ventrolateral medulla (Ellacott and Cone, 2004; Benarroch, 2005; Andresen et al., 2007a; Rinaman, 2007; Ulrich-Lai and Herman, 2009). Different projection neurons feature frank excitability differences in their ion channel expression which differently tune their action potential outputs along the pathway (Bailey et al., 2007) and these ion channels often may be the postsynaptic targets of neurotransmitter regulation through metabotropic receptors (see below). Although some of these NTS projection neurons are second order, some belong to a relatively rare class, higher order neurons in the NTS that lack direct ST contacts (Bailey et al., 2006a, 2007). Whether second order or higher order, ultimately the flow of cranial visceral afferent information results in autonomic regulatory pathways of the sympathetic and parasympathetic nervous systems. As short as many pathways through NTS can be, it is clear that key integration occurs at the first synapse, i.e., the afferent synapse.

## Presynaptic Cranial Afferent Terminals

Over 75% of all neurons in the medial NTS are second order neurons (McDougall et al., 2009; Andresen et al., 2012; McDougall and Andresen, 2012). Since the A/C axon distinction extends to central synapses within the NTS, >80% of all vagal afferent terminals are likely C-type (Aicher et al., 1999; Andresen et al., 2012). TRPV1 reliably marks C-type ST afferent inputs but is never expressed postsynaptically, i.e., within the NTS neuron (Doyle et al., 2002; Jin et al., 2004b; Andresen et al., 2007b, 2012; Andresen and Peters, 2008). Nodose neurons share similar biophysical distinctions in ion channels and excitability as occur in dorsal root ganglion neurons. C-type TRPV1+ nodose neurons express TTX-resistant sodium channels and have characteristically prolonged action potentials in their cell bodies along with substantial other differences in ion channel biology compared to A-type nodose afferent neurons including potassium channels (Schild et al., 1994; Schild and Kunze, 1997; Li and Schild, 2007; Li et al., 2007, 2011; Zhou et al., 2010). As a result, C-type neurons display much stronger frequency dependent action potential broadening which limits repetitive excitation.

## Mechanism in Common – Synchronous Glutamate Transmission

A- and C-type primary afferents have substantially different action potentials and biophysical excitability (Schild et al., 1994; Schild and Kunze, 2012). Centrally, action potentials activate fast synaptic transmission by triggering voltage-activated calcium channels. This calcium entry mediates fast, synchronized fusion of glutamate vesicles. Despite characteristic differences in afferent excitability, fast glutamate transmission is remarkably uniform across all ST afferents (Bailey et al., 2006b; Andresen and Peters, 2008; Peters et al., 2008; Jin et al., 2010). Activation of a single ST afferent axon reliably triggers a highly synchronous release of glutamate vesicles from all ST afferents with nearly zero failures. This synchronous glutamate activates excitatory postsynaptic currents (EPSCs) via postsynaptic AMPA receptors (Hermes et al., 2008). Antagonists to non-NMDA receptors fully blocked ST evoked EPSCs in intracellular recordings from second order NTS neurons and NMDA antagonists alone were without effect (Andresen and Yang, 1990; Doyle et al., 2002; Kline et al., 2007; Zhang and Mifflin, 2007). These ST-EPSCs arrive at very consistent latencies measured as low jitter (SD of latency of <200 μs) that indicate monosynaptic connections (Doyle and Andresen, 2001; Andresen and Peters, 2008). Incorporation of afferent dye labeling in such experiments show that dye placed on peripheral cranial afferent axon trunks was transported centrally and appeared as multiple fluorescent puncta contacting NTS neurons and identifying them anatomically as second order neurons (Mendelowitz et al., 1992; Balkowiec et al., 2000; Doyle and Andresen, 2001; Kline et al., 2002; Chen and Bonham, 2005; Zhang and Mifflin, 2007; Andresen and Peters, 2008). Such dye identified neurons showed afferent boutons on or near the cell body (a somatodendritic pattern) and when tested in brain slices evoked similarly low jitter EPSC responses. Remote ST activation rarely activates NMDA receptors even using rapid bursts of ST shocks in low magnesium conditions (Jin et al., 2003). In transverse NTS slices, NMDA receptor mediated synaptic responses were reported in 3–4-week-old animals (Aylwin et al., 1997; Baptista et al., 2005a) or a subset of second order, adult NTS neurons with myelinated afferents (Jin et al., 2003). In transverse slices, the ST and recipient neurons are in close proximity (often 200–300 μm) but the contributions of the spread of the electrical stimulus beyond the ST or the developmental state of the animals are difficult to assess. Certainly, NMDA receptors were present on some neurons since exogenous NMDA selective agonists activated small inward currents (Drewe et al., 1990) but, in many neurons, neither NMDA nor massive glutamate release with capsaicin evoked currents under non-NMDA blockade (Doyle et al., 2002). Thus, the participation of NMDA receptors in ST afferent transmission has been complicated and controversial (Baude et al., 2009). Together these findings suggested that NMDA receptors were located extrasynaptically and out of reach of synaptically released glutamate on second order NTS neurons (Doyle et al., 2002). This suggestion is supported by ultrastructural studies that showed NMDA receptors mostly localized to extrasynaptic membranes rather than within asymmetrical synapses adjacent to vagal afferent terminals (Aicher et al., 1999). Interestingly, these same structural studies indicated the common presence of presynaptic NMDA receptors on vagal afferent terminals (Aicher et al., 1999), although no functional studies have indicated their role. Thus, fast synaptic transmission generates large reliable EPSCs for all ST afferent fibers with similar frequency dependent depression for both TRPV1+ and TRPV1− afferents. This frequency dependent depression results to some degree from vesicle depletion as well as other autoreceptor feedback mechanisms.

One difference in fast glutamatergic transmission that routinely distinguished C-fibers ST transmission was their strong tendency to fail in a use-dependent manner. TRPV1−, A-type ST afferents rarely failed. On a single shock of activation, TRPV1+ and TRPV1− afferents failed on average less than 1% of trials. At high frequencies of activation, failure rates (missing EPSCs per stimulus shock) approached rates of 30% for C-type afferents while remaining below 5% for A-type afferents (Andresen and Peters, 2008). Thus, the basic mechanisms of synchronous glutamate transmission are similar but it may be that ion channel differences impact the invasion of terminals or their central conduction that fails more often in C-type afferents than A-type afferents. The dynamics of synaptic failures may indicate successful invasion of the synaptic terminal and may occur distally and outside of the electrotonic influence of the terminal (Bailey et al., 2006b; McDougall et al., 2009). Since the two afferent fiber types and their excitability are intrinsically distinct in many ways, the use-dependent failures most likely reflects the behavior of those ion channels in regenerative spike generation (Schild et al., 1994; Schild and Kunze, 1997; Li and Schild, 2007; Li et al., 2007). These differences between conduction and vesicle release are important to understanding receptor mediated mechanisms and sites of modulation.

Detailed analyses of low jitter ST-EPSCs indicated that they arose from an average of 18–25 active contacts that form the EPSC (Bailey et al., 2006b; Andresen and Peters, 2008; Peters et al., 2008). Such a functioning array of multiple contacts is quite consistent with labeling studies of ST afferents which showed varicosities resembling multiple release sites distributed along single axons (Anders et al., 1993; Kubin et al., 2006). The redundant contact pattern requires release sites formed by en passant contacts from each afferent axon and the multiple release sites generate the very large and reliable safety factor of transmission between single afferents and the second order neuron. The probability of synchronous glutamate vesicle release from a readily releasable pool averaged a surprisingly high 90% in an external calcium concentration of 2 mM. However, the amplitudes of these ST-EPSCs decreased substantially when activation frequencies were above 50 Hz – a physiologically modest frequency for naturally activated myelinated afferents but a near maximal frequency for typical C-fiber afferents. Remarkably however, the key features of fast synchronous glutamate transmission were indistinguishable between A- and C-type cranial afferents. This synchronous glutamate mechanism reliably evokes action potentials in the postsynaptic neuron and presumably conducts this information beyond the NTS up to frequencies of about 20 Hz before the EPSC depresses to become less reliable at higher frequencies (Andresen and Yang, 1995; Schild et al., 1995; Liu et al., 1998).

## Mechanism of Distinction – TRPV1-Operated Glutamate Release

Given the fundamental similarities in synchronous glutamate release, a surprising difference has emerged between TRPV1+ and TRPV1− second order NTS neurons regarding a different mode of glutamate release – basal or spontaneous glutamate events. Spontaneous release has long been thought to result from stochastic, rare spontaneous fusions from the readily releasable pool of vesicles – that is vesicles staged and primed for release (Katz, 1971; Chung and Kavalali, 2006; Kavalali et al., 2011; Ramirez and Kavalali, 2011). The similarities in fast glutamatergic excitatory transmission between A- and C-type ST afferent inputs may have delayed recognition of what in hindsight should have been obvious (Peters et al., 2010, 2011; Shoudai et al., 2010). NTS neurons with C-type afferents experienced a 5–10-fold higher spontaneous rate of EPSC activity. These high rates of EPSCs in TRPV1+ NTS neurons were unrelated to afferent activity since they persisted when action potentials were blocked by TTX. In fact, high rates of action potential driven activity caused depressed synchronous release but spontaneous release was elevated suggesting that two pools of different vesicles were responsible (Peters et al., 2010, 2011; Shoudai et al., 2010). Antagonism of TRPV1 (e.g., SB-366791) decreased spontaneous release at NTS neurons with TRPV1+ ST afferents without altering the amplitude of synchronous EPSCs. In addition, the spontaneous or miniature (in TTX) release rate closely followed changes in temperature between 30 and 37°C suggesting that temperature effectively gated presynaptic TRPV1 receptors. Thus, TRPV1 in ST terminals actively drove the release of glutamate vesicles at physiological temperatures and violates the canonical view that TRPV1 receptors have thermal gating thresholds of >43°C (Julius and Basbaum, 2001). Preliminary work indicates that both asynchronous release and thermally evoked release are attenuated in TRPV1 knockout mice (Peters, 2011). Activation of TRPV1 may depolarize synaptic terminals through its cation selectivity and inward current, a process that can activate voltage-activated calcium channels to release glutamate. The cation selectivity of TRPV1 constitutes a direct pathway for the influx of calcium ions into these terminals (Caterina et al., 1997). Blockade of voltage-activated calcium channels with cadmium did not alter spontaneous rates of EPSCs but blocked ST evoked synchronous EPSCs suggesting that TRPV1 provided the calcium for spontaneous vesicle release (Fawley et al., 2011). In the presence of cadmium, thermally evoked increases in mEPSCs persisted (Fawley et al., 2011). Overall, ST TRPV1 is quite active within a range of physiologically relevant temperatures and TRPV1-operated glutamate release represents a novel and tonic signaling mechanism that releases neurotransmitter even in the absence of afferent action potentials. It is particularly interesting that two other ligand-gated ion channels are expressed on ST terminals – the serotonin gated channel, 5-HT3, and the purinergic P2X3 channel – and in many respects these channels resemble TRPV1 signaling in being permeable to calcium and capable of influencing vesicular neurotransmitter release (Glaum et al., 1992; Doucet et al., 2000; Jin et al., 2004b; Mazda et al., 2004; Wan and Browning, 2008; Cui et al., 2012b). However, neither 5-HT3 nor P2X3 appears to influence basal release tonically, a point which further distinguishes TRPV1 as a tonic signal that does not require neural activation.

A-type cranial visceral afferents generate synchronous EPSCs but have very low basal spontaneous glutamate release. In contrast, C-type afferents have similar synchronous EPSCs, but release substantial numbers of glutamate vesicles spontaneously. Activation of action potential driven synchronous EPSCs enhanced the TRPV1-operated vesicle release for a period of seconds and produced asynchronous release sufficient to trigger additional action potentials in the postsynaptic neuron (Peters et al., 2010, 2011; McDougall and Andresen, 2013) in basal conditions that are incremented even higher in an asynchronous mode of release. The physiological meaning of the TRPV1-operated pool of vesicles is only beginning to be appreciated. This TRPV1-operated pool has been implicated as potentially playing a role in the laryngeal chemoreflex and the exaggerated apnea in animal models of Sudden Infant Death Syndrome (Curran et al., 2005; Xia et al., 2007, 2008, 2010, 2011). More comprehensive investigations are needed to fully evaluate this new form of afferent synaptic transmission and its implications in multi-system homeostatic regulation.

## Metabotropic Signaling

Ionotropic receptors for glutamate and GABA are the mainstay for excitatory and inhibitory transmission (respectively), within the NTS. Integration, however, even at second order neurons is highly differentiated through both homo- and heterosynaptic interactions by glutamate and GABA. With multiple “accessory” transmitters present in the NTS (especially peptides, see above), the interaction potential blossoms and assessments require quite elaborate testing to establish cause and effect. Much of this interaction utilizes metabotropic receptors which employ second messenger signaling (Conn, 2003). Metabotropic glutamate receptors, mGluRs, powerfully modulate both ST afferent glutamate release as well as GABA release by presynaptic mechanisms (Glaum and Miller, 1993; Foley et al., 1999; Hay et al., 1999; Hoang and Hay, 2001; Chen et al., 2002; Jin et al., 2004a; Browning et al., 2006; Browning and Travagli, 2007; Austgen et al., 2009; Fernandes et al., 2011). These presynaptic effects are heterogeneous and, depending on the subunit composition, can be negative or positive regulators of neurotransmitter release even across otherwise similar second order NTS neurons. In addition, mGluRs can modify postsynaptic current to alter NTS neuron excitability (Sekizawa and Bonham, 2006). Little system specific information exists for these interactions in the NTS particularly regarding which visceral afferents are involved or the specific destinations of projections out of the NTS. The TRPV1-operated pool presents a new challenge since pharmacological assays of spontaneous or miniature glutamate events may not be representative of synchronous or afferent driven mechanisms (Fawley et al., 2011). Because these receptors are present in multiple pathways, functionally important interactions may be triggered during drug interventions in the NTS. Therefore, differential expression of specific GPCRs may help to specifically tune individual pathways. Presynaptic mGluRs are common GPCRs in the NTS (Hay et al., 1999; Jin et al., 2004a; Fernandes et al., 2011) and presynaptic expression of GPCRs for peptides common to peripheral or forebrain (e.g., hypothalamus) neuron sources distinguishes ST transmission at many neurons. These peptide GPCRs are primarily presynaptic on ST afferents but generally have much more limited representations. GPCRs for angiotensin (AT1), vasopressin (V1a), OT, opioid (MOR), ghrelin (GHSR1), and CCK differentially control glutamate release on limited subsets of neurons. No clear associations have been established between particular GPCRs and different afferent modalities or their myelination. Little is known about the access of these GPCR signal transduction to the TRPV1-operated mode of glutamate release.

## Novel NTS-TRPV1 Signaling

Fat and membrane lipid metabolites are newly appreciated as CNS signaling molecules (Pingle et al., 2007; Scherer and Buettner, 2009; DiPatrizio and Piomelli, 2012). Lipoproteins and metabolites including anandamide (AEA) likely act as endocannabinoid and/or endovanilloid signaling molecules that alter reflex function when introduced into the NTS (Geraghty and Mazzone, 2002; Brozoski et al., 2005, 2009; Seagard et al., 2005). Co-expression of the cannabinoid receptor (CB1) and TRPV1 is common in dorsal root ganglion neurons (Amaya et al., 2006; Binzen et al., 2006). CB1 and TRPV1 interact with lipid derived mediators such as arachidonate, 2-arachidonoylglycerol, AEA, eicosanoids, epoxyeicosanoids, and prostaglandins. AEA was detected in the dorsal vagal complex samples which included the NTS (Seagard et al., 2004; Chen et al., 2009) and the AEA content in the NTS region increased when blood pressure was raised (Seagard et al., 2004). However, the biochemical pathways for genesis of potential lipid mediators are complex and interconnected so that the ultimate active compounds and cellular signaling are often difficult to establish (Buczynski and Parsons, 2010; Katona and Freund, 2012). Depolarization of NTS neurons released an endocannabinoid that controlled GABA release via CB1 (Chen et al., 2010). Although CB1 generally inhibits neurons and neurotransmitter release (Derbenev et al., 2004), AEA not only inhibited glutamate release via CB1 activation but also enhanced release via TRPV1 activation – dual opposing actions on a common biological process (Tognetto et al., 2001). Experimental use of plant derived agonists and endogenous ligands may be confounded by this TRPV1-CB1 interaction. *N*-Arachidonoyl-dopamine (NADA) is an endogenous “capsaicin-like” substance that can act at TRPV1 (Huang et al., 2002). Such paradoxical actions of substances which crossover to activate both CB1 and TRPV1 are not well understood especially in systems in which these receptors are co-localized including primary afferents (Ahluwalia et al., 2000; Price et al., 2004). Lipids together with various inflammatory cytokines are linked to the NTS and correlated to the development of chronic hypertension and obesity in humans (Chae et al., 2001; Katagiri et al., 2007) and in animal models (Waki et al., 2008; Wang and Wang, 2009; Takagishi et al., 2010). For example, systemic injection of lipopolysaccharide or interleukin-1β triggered sustained glutamate release from the NTS that was prevented by TTX (Mascarucci et al., 1998). Prostaglandin E2 depresses only TRPV1+ ST evoked EPSCs in NTS neurons (Laaris and Weinreich, 2007). Cannabinoids are implicated in central plasticity and in cyclooxygenase metabolism to prostaglandins (Yang and Chen, 2008; Heifets and Castillo, 2009). Thus, the potential for signaling crossover between CB1 and TRPV1 is considerable, complex, and poorly understood.

TRPV1-operated glutamate mechanism offers a new focal point for the integration of multiple signals with substantial clinical importance. Intriguing clinical and basic observations point to multiple risk factors for multi-organ system pathology that relate directly or indirectly to cranial afferents, TRPV1 and NTS signal processing. One extrapolation of these complex correlations concerns inflammation, a well recognized co-factor in multiple disease processes that impact homeostatic systems. In spinal dorsal horn, TRPV1 is part of a chronic pathological transformation of afferent signaling (allodynia/hyperalgesia; Holzer, 2004) but whether analogous changes occur in the NTS are unknown. Markers of inflammation, e.g., interleukin-6, are positively correlated with increases in blood pressure (Chae et al., 2001), stress, and hypothalamic-pituitary-adrenal axis activation (Serrats et al., 2010) with elevated morbidity (Katagiri et al., 2007) and TRPV1 targeted drugs have been touted as anti-inflammatory agents (Tsuji et al., 2010). Inflammation within cardiovascular areas of the NTS is reported in neurogenic hypertensive rats (Waki et al., 2008). Interestingly, inflammatory cytokine responses in induced dietary salt linked forms of hypertension are modified in TRPV1 knockout mice (TRPV1^−/−^; Wang and Wang, 2009). TRPV1^−/−^ mice are reported to be resistant to obesity from high fat diets that induce low level inflammation (Motter and Ahern, 2008). Such associations and roles for peripheral vs. central TRPV1 receptors are poorly understood. Thus, the deficit in information regarding C-fibers and TRPV1 in the NTS has broad potential implications that are critical to understanding pathophysiology (Szallasi et al., 2007; Wong and Gavva, 2009).

## Conflict of Interest Statement

The authors declare that the research was conducted in the absence of any commercial or financial relationships that could be construed as a potential conflict of interest.
